# Web 2.0 Chronic Disease Self-Management for Older Adults: A Systematic Review

**DOI:** 10.2196/jmir.2439

**Published:** 2013-02-14

**Authors:** Michael Stellefson, Beth Chaney, Adam E Barry, Enmanuel Chavarria, Bethany Tennant, Kim Walsh-Childers, P.S Sriram, Justin Zagora

**Affiliations:** ^1^Center for Digital Health and WellnessDepartment of Health Education and BehaviorUniversity of FloridaGainesville, FLUnited States; ^2^Department of JournalismUniversity of FloridaGainesville, FLUnited States; ^3^Department of MedicineUniversity of FloridaGainesville, FLUnited States; ^4^Department of Health Education and BehaviorUniversity of FloridaGainesville, FLUnited States

**Keywords:** chronic disease, self-care, internet, social media

## Abstract

**Background:**

Participatory Web 2.0 interventions promote collaboration to support chronic disease self-management. Growth in Web 2.0 interventions has led to the emergence of e-patient communication tools that enable older adults to (1) locate and share disease management information and (2) receive interactive healthcare advice. The evolution of older e-patients contributing to Web 2.0 health and medical forums has led to greater opportunities for achieving better chronic disease outcomes. To date, there are no review articles investigating the planning, implementation, and evaluation of Web 2.0 chronic disease self-management interventions for older adults.

**Objective:**

To review the planning, implementation, and overall effectiveness of Web 2.0 self-management interventions for older adults (mean age ≥ 50) with one or more chronic disease(s).

**Methods:**

A systematic literature search was conducted using six popular health science databases. The RE-AIM (Reach, Efficacy, Adoption, Implementation and Maintenance) model was used to organize findings and compute a study quality score (SQS) for 15 reviewed articles.

**Results:**

Most interventions were adopted for delivery by multidisciplinary healthcare teams and tested among small samples of white females with diabetes. Studies indicated that Web 2.0 participants felt greater self-efficacy for managing their disease(s) and benefitted from communicating with health care providers and/or website moderators to receive feedback and social support. Participants noted asynchronous communication tools (eg, email, discussion boards) and progress tracking features (eg, graphical displays of uploaded personal data) as being particularly useful for self-management support. Despite high attrition being noted as problematic, this review suggests that greater Web 2.0 engagement may be associated with improvements in health behaviors (eg, physical activity) and health status (eg, HRQoL). However, few studies indicated statistically significant improvements in medication adherence, biological outcomes, or health care utilization. Mean SQS scores were notably low (mean=63%, SD 18%). Studies were judged to be weakest on the Maintenance dimension of RE-AIM; 13 reviewed studies (87%) did not describe any measures taken to sustain Web 2.0 effects past designated study time periods. Detailed process and impact evaluation frameworks were also missing in almost half (n=7) of the reviewed interventions.

**Conclusions:**

There is need for a greater understanding of the costs and benefits associated with using patient-centered Web 2.0 technologies for chronic disease self-management. More research is needed to determine whether the long-term effectiveness of these programs is sustainable among larger, more diverse samples of chronically ill patients. The effective translation of new knowledge, social technologies, and engagement techniques will likely result in novel approaches for empowering, engaging, and educating older adults with chronic disease.

## Introduction

### Background

According to the Centers for Disease Control and Prevention, nearly half of all adults in the United States are living with at least one chronic health condition [1]. Globally, chronic diseases such as heart disease, chronic respiratory illness, and diabetes are by far the leading cause of death, topping most all-cause morbidity lists [2]. As a result, chronic disease care accounts for eighty cents of every health care dollar spent (80%) of total health care expenditures [1]. By 2015, it is estimated that 7 of every 10 (70%) adults aged 50-64 will have been diagnosed with at least one chronic condition, with nearly half living with two or more chronic conditions [3].

Among individuals with chronic disease, the use of the Internet as a “first stop” for health information has increased steadily [4]. Even after controlling for various demographic factors such as age and education, Internet users living with chronic disease are slightly more likely than other Internet users to access health information online and more likely to share acquired health information with others [5]. Considering that more than 2 of every 4 (50%) adults aged 65 and older are now using the Internet or email [6], and 7 in 10 (70%) Internet users 65 and older go online daily [6], Internet-mediated chronic disease self-management and self-monitoring interventions may exhibit great potential to reach a broad population of chronically ill older patients [7-9].

### Chronic Disease Patients and Social Networking

Older adults remain strongly connected to offline sources of medical assistance and advice [5]. This is especially true for Americans 65 and older, of whom only 53% used the Internet and only 34% used any social networking site as of spring 2012 [10]. An earlier report in 2011, however, showed Internet use to be significantly more common among adults 50-64 years old, with 78% online, 58% seeking health information on the Web, and 47% using social networking sites [11]. Research shows that the most common explanation offered by those who do not use the Internet is the perception that the Internet is irrelevant to them; they can meet information and communication needs in other ways and see no point in going online [12]. Among older adults, another major reason for less frequent use of the Web, including social media, is lack of access to high-speed Internet connections. While 60% of adults aged 50-64 have broadband access at home, the figure falls to 30% for those 65 and older [12]. Blogging and online health discussion forums are the two most popular social networking activities for people living with chronic disease, primarily because these activities allow an Internet user to dive deeply into a health topic without the need for advanced technical knowledge sometimes associated with using social media/networking applications such as Twitter or Facebook [5].

Nonetheless, use of social networking among older adults is growing [6]. Approximately 1 in 3 (34%) older Internet users access social networking sites like Facebook and LinkedIn, and approximately 1 in 5 (20%) of these users contributes to these sites regularly by tagging, categorizing, or commenting on online health/medical content [5,6]. Patients increasingly have begun to use the Web as a communication tool, instead of simply an information vending machine [13]. Older adults, in particular, are willing to share self-care information within selected social networks for the purpose of giving and receiving disease-specific self-management information [14]. The evolution of e-patient communities has led to greater opportunities for knowledge acquisition and social support, leading to improved health-related quality of life (HRQoL) [7-9,15,16].

### Transitioning from Web 1.0 to Web 2.0 in Chronic Disease Management

Traditionally, public health experts have provided chronic disease information in static form through Web 1.0 interventions, which primarily make written and audio materials available online [17-19]. The use of these eHealth interventions has shown potential to improve health outcomes cost-effectively [15,20-23]. The rapid growth in adoption of Web 2.0 technologies, as documented above, suggests that participatory Internet interventions can help older individuals with chronic diseases become actively engaged in their own health care [15,20,21]. Controlling for age, education, and type of Internet access, living with chronic disease increases the likelihood of contributing to or consuming user-generated health content such as blog posts, hospital or doctor reviews, and podcasts [5,24]. Moreover, online discussion boards provide an open-access space for chronic disease patients to exchange information and learn about how to control disease exacerbations [25-28]. Additionally, available evidence shows that online self-help groups can enhance social capital in ways that do not undermine, and might in some cases strengthen, hyperpersonal connections between patients and providers [29-31].

Multimedia-sharing software enables chronic disease patients to share disease management videos, wikis, and podcasts without the need for advanced technical knowledge. Teleconferencing tools such as Skype provide intimate, two-way communication channels for patients and providers to share information, provide emotional support, and offer practical disease management advice from a distance [32]. These types of social software promote collaboration between patients, caregivers, and practitioners, leading to marked shifts in how patient education for chronic disease management [33].

### Current Investigation

Given that older adults suffering from chronic disease are becoming more likely participate in Web 2.0 e-patient communication, it is surprising to note the paucity of formal evaluations examining use of Internet-mediated information and communication technologies (ICTs) among older adults. Specifically, there are no review articles that investigate the planning, implementation, and/or effectiveness of Web 2.0 self-management interventions among older adults with various chronic diseases. Consequently, few evidence-based recommendations exist regarding the development of Web 2.0 interventions for this vulnerable population [34]. A synthesis of the empirical evidence regarding the benefits and limitations of Web 2.0 interventions can enhance the transferability and translational potential of participatory technologies designed for healthy aging. Because of this emergent need, the primary objective of this study was to systematically review the planning, implementation, and overall effectiveness of Web 2.0 chronic disease self-management interventions delivered to older adults living with chronic disease.

## Methods

### Overview

First, it is important to operationalize several terms that informed our literature screening process. *Web 2.0* was defined as the technical, aesthetic, and functional criteria established to enable collaboration and sharing of information between users on the Internet [35]. A *chronic disease* was defined as a prolonged illness not resolving spontaneously or becoming cured completely, causing nonreversible pathological alteration and residual disability [1]. We specifically searched for studies examining selected chronic diseases (eg, heart disease, chronic obstructive pulmonary disease (COPD), arthritis, hypertension and diabetes) known to be pervasive worldwide [2]; however, studies of individuals with other chronic diseases were included if they met other search criteria. A c*hronic disease self-management intervention* was defined as a program specifically designed to train patients to live with their chronic condition by teaching them behaviors to promote self-care and/or foster self-confidence in long-term self-management capability [36,37].

### Search Procedures

Because Web 2.0 formally emerged in the research literature in 2004 [38], only manuscripts published in English from January 2004 to October 2012 were considered. The searched databases included: ERIC, PsychINFO, PubMed, Academic Search Premiere, CINAHL Plus, and Applied Social Sciences Index and Abstracts. Search methodology included entering various combinations of key search terms into each database, using controlled vocabulary with the Boolean operators AND and OR. The search terms included: *chronic disease, chronic illness, heart disease, diabetes, arthritis, hypertension, COPD, self-care, self-management, outcome, internet,* and *website*. The terms “older adult” or “elderly” were not included as search terms to prevent unintentional exclusion of studies examining adults aged 50 and older, the age cutoff previously used by international health organizations [39]. Following the literature search, reference lists for each eligible study were reviewed for additional articles.

### Selection Criteria

The experimental unit of analysis in this review was studies of Web 2.0 interventions administered to adults 50 and older (mean age ≥ 50), living with one or more chronic disease(s). Articles had to describe the planning, implementation, and impact of the intervention by measuring either process (eg, attitudes, self-efficacy, social support), functional (eg, health behavior, participants’ subjective experience of functioning), and/or clinical (eg, morbidity, mortality, HRQoL) outcomes. To minimize the risk of assessment bias, 2 trained researchers documented the literature search at each step of the screening process by tracking results generated within each database search. Titles and abstracts of each study were assessed independently by 1 reviewer and checked by the second. Disagreements between reviewers were discussed and resolved by consensus after referring to the search protocol. A third qualified reviewer in eHealth communication was consulted to resolve any discrepancies before data were extracted.

### Study Quality Score

We also assessed the extent to which each study minimized bias and maximized internal and external validity to obtain an indicator of study quality using the RE-AIM evaluation framework [40]. The extracted data from the retained studies were evaluated according to the five dimensions of RE-AIM: Reach, Efficacy, Adoption, Implementation, and Maintenance [40]. *Reach* refers to the percentage and risk characteristics of individuals who participate in an intervention and how representative they are of the population being considered. *Effectiveness* concerns both the intended or positive outcomes of an intervention on targeted outcomes (eg, process, functional, and clinical) and the possible negative or unintended consequences on quality of life and nontargeted outcomes. *Adoption* is characterized as the participation rate and representativeness of both the settings in which an intervention is conducted (eg, doctor’s offices, communities) and the intervention staff who deliver the intervention (eg, physicians, health educators). *Implementation* refers to the extent to which an intervention is delivered consistently across different components of staff over time. *Maintenance*, at the individual level, describes the long-term results of an intervention (≥6 months following intervention contact) among participants; at the setting level, it refers to either the short-term continuation or long-term institutionalization of an intervention once the research project and its supports are withdrawn [41].

RE-AIM can help media developers create practical products that are more likely to be widely adopted, feasible in medical practice, and able to produce public health impact. The framework has been successfully applied to evaluate the impact of interactive technology approaches [42]. For example, a focus on the reach of individuals who engage with technology and the robustness of intervention effects is crucial to designing self-management support systems that use appropriate multimedia aids to help all patients, particularly those from low-literate populations. In addition, self-management support is enhanced by focusing on factors such as adoption, implementation, and sustainability to provide actionable information [42].

To develop a unique assessment instrument for this evaluation task, we assembled quality items from a number of systematic review guides [43-46]. Reach was assessed by analyzing the representativeness of our sample by evaluating the sampling frame, screening criteria, and response rate using items from the *Guide to Community Preventive Services: Systematic Reviews and Evidence-Based Recommendations* [43] and the Effective Public Health Practice Project [44]. To assess efficacy, we used a variety of validated items [43-46] to assess the suitability of study design, credibility of data collection, program evaluation, and statistical analyses. We also created two new items to evaluate how missing data were handled as well as whether *P* values and effect sizes were reported.

Adoption was assessed at both the setting and staff levels. At the setting level, we assessed the short-term feasibility of delivering the program and whether the Web 2.0 intervention was incorporated into the existing structure of the sponsoring institution or organization. At the staff level, we assessed if the project manager possessed adequate expertise and whether stakeholder feedback was collected among program staff members. Implementation was assessed by evaluating Web 2.0 uptake to determine web accessibility, participation adherence [44], and duration (dosage) and intensity of participant exposure to the Web 2.0 intervention. In addition, to assess intervention development and program integration, we administered items from the Intervention Development and Implementation subscales of the Preffi 2.0 health promotion quality assessment package [46], along with one item we created for evaluating use of incentives (eg, gift cards) for participation. Maintenance was assessed at the setting and participant levels. At the setting level, we assessed the contextual conditions and long-term feasibility of each reviewed study using the Contextual Conditions and Feasibility subscale of the Preffi 2.0 package [46]; we added one item to determine whether policies were developed to institutionalize Web 2.0 in practice. At the participant level, we created new items based on RE-AIM evaluation criteria [41] to assess whether positive intervention effects were observed at ≥ 6 months or ≥ 1 year. We also assessed whether long-term attrition remained at or below 30% at follow-up.

Multimedia Appendix 1 lists of all quality criteria measures (with scale origins and ranges) that were used to evaluate each study, and Multimedia Appendix 2 lists the actual items (with response options and codes) organized by RE-AIM dimensions. Overall, there were 38 total items programmed into an online data extraction rubric that was built to input data from this quality assessment. This tool was pilot tested by the research team on one manuscript that was not included in the final group of reviewed studies. Following the pilot test, minor modifications were made to the format and wording to improve clarity and accuracy. Scores on these items were summed to compute a raw study quality score (SQS) (range = 1 to 61) for each retained study. To interpret this aggregated total score, each raw score was transformed by dividing it into the total possible score (61) and then multiplying it by 100 to obtain a percentage score for each study that ranged from 0% to 100%. Higher percentage scores on the SQS were indicative of higher quality study design.

The research team also classified Web 2.0 implementation characteristics including design (ie, technical, information architecture, aesthetic, and functional), interactivity (ie, synchronous or asynchronous communication), and content (ie, disease management information, web content) described within each reviewed study. We then analyzed which Web 2.0 intervention qualities were associated with targeted outcomes (eg, process, functional, and clinical) and possible negative or unintended consequences of the intervention on HRQoL and nontargeted outcomes.

## Results

### Study Characteristics

**Figure 1:**
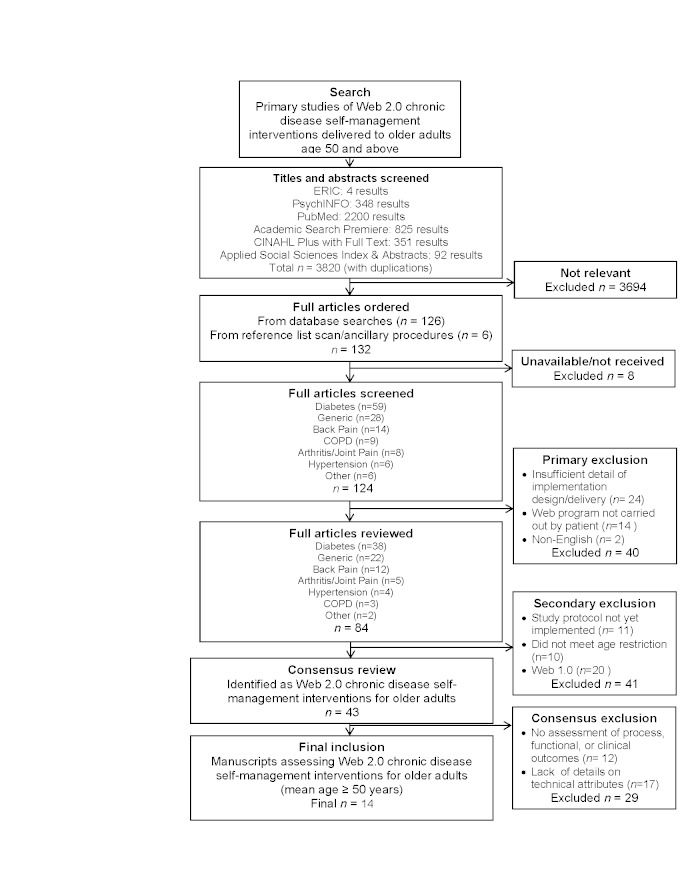
Stem-tree illustrating manuscript selection process using various search databases and combinations of controlled vocabulary.

Figure 1 illustrates the three-round process used to select articles from the initial pool of 3820 articles identified. Eliminating manuscripts that were not relevant (n=3694) left 126 articles with another 6 identified through reference list scans. Eight articles were unavailable through the institutional e-library database leaving 124 to screen. During the initial review, articles were excluded for these reasons: insufficient details on research design and delivery (n=24); web program not being used by a chronic disease patient (n=14); or manuscripts written in a language other than English (n=2). The secondary wave of exclusion was completed following a full-text review of the remaining 84 articles. Forty-one of these articles were deemed ineligible due to reporting incomplete research protocols (n=11), participants not meeting the pre-specified age range (n=10), or because they involved only a noninteractive Web 1.0 interface (n=20). The secondary exclusion left 43 articles for final review. The final wave of exclusion resulted in 28 additional articles being excluded because they lacked any process, functional or clinical outcome assessments (n=11), or because they provided little detail on Web 2.0 components included within the intervention (n=17). These procedures produced 15 articles that met our inclusion criteria.

The final sample of studies were published in a variety of peer-reviewed journals, including the Journal of Medical Internet Research [47-51], CIN: Computers, Informatics, Nursing [52], Telemedicine and e-Health [53,54], Diabetes Care [55,56], The Diabetes Educator [57], Patient Education and Counseling [58], Arthritis & Rheumatism [59], Health Education and Behavior [60], and Journal of Pain and Symptom Management [61]. Included articles had been published between 2005 and 2012, with the majority (n=10 or 67%), published in 2010 or later.

Multimedia Appendix 3 describes the primary characteristics of interest (authorship, study purpose, sample size, and RE-AIM attributes) in each retained study. Multimedia Appendix 4 reports the SQSs for each reviewed study on each RE-AIM dimension and subdimension. On a scale from 1 to 61, the raw SQSs for all reviewed studies ranged from 16 (26%) to 52 (85%). The mean raw SQS score of all reviewed studies was 38.33 (SD 10.43), which corresponded to a mean SQS score of 63% (SD 18%), which was notably low. Only four studies [51,56,58,59] scored 80% or above on the SQS scale.

 In the following, we report on results from the reviewed studies with respect to each aspect of the RE-AIM framework.

### Reach Characteristics

#### Representativeness

Eight studies examined individuals with diabetes [48-51,55-58], with four (n=4) studies specifically targeting type 2 diabetes [49,51,56,58]. Two studies examined individuals with COPD [47,61], and two investigated patients with arthritis or related musculoskeletal disorders [54,59]. Three (n=3) other studies were designed for individuals suffering from one or more chronic condition(s) [52,53,60]. The mean SQS score on the Reach dimension was 2.33 (SD 0.62) on a scale from 0 to 3.

#### Participation Rate and Country

Sample sizes ranged from n=18 participants in a qualitative feasibility study [53] to n=855 participants in one randomized controlled trial (RCT) [59]. Eight studies had more than 250 participants, while six studies reported n≤100. Eleven of the reviewed studies enrolled primarily White participants, ranging from 67% (181 of 270 participants) to 97% (38 of 39 participants) of total sample sizes. A little more than half of the reviewed studies (n=8) consisted of mainly White females [48,50,52,53,56,57,59,60]. Across all reviewed studies, the mean age of participants ranged from 52 to 69 years. Studies took place in several countries; twelve in the United States, and one each in Canada, Australia, and the Netherlands.

### Empirical Effectiveness

#### Theoretical Framework and Research Design

Eleven (n=11) studies were RCTs [47-50,52,55-58,60,61], while five adopted a randomized cluster [54], quasi-experimental [60], cross-sectional [51], or qualitative [53] design. Constructs from the social cognitive theory (eg, self-efficacy) were used in eight studies [47,50,54,56,57,59-61], while the social ecological theory and the 5 As (assess, advise, agree, assist, and arrange) self-management model were used in two studies [49,58]. Four reviewed studies did not specify a theoretical framework [48,52,53,55]. The reviewed studies had relatively stronger mean SQS scores on suitability of study design (mean=5.47, SD 1.64 on a scale from 1 to 7) versus overall program evaluation (mean=7.2, SD 2.98 on a scale from 0 to 12).

#### Process Outcomes

A variety of process outcomes were measured in the reviewed studies, providing evidence that Web 2.0 improved confidence in several aspects of self-management. In five studies, use of Web 2.0 interventions was associated with statistically significant improvements in self-management self-efficacy [47,56,57,59,60], with one study noting positive trends falling just short of statistical significance (*P*=.06) [56]. Four studies reported positive responses towards using Web 2.0 for communicating with health care providers (ie, nurses, care managers) [51-54], and five reviewed studies showed improvement in perceived social support [48,50,56,57,61].

Chronic disease patients’ utilization of Web 2.0 self-management features was also widely examined in 14 of the 15 studies. To determine which Web 2.0 features were accessed most/least often, web log activity was mined in 13 studies [47-52,54-56,58-61]. Three studies [49,51,58] explicitly noted that individuals enrolled in Web 2.0 interventions at baseline did not participate after the first few weeks. For example, in a 4-month study of individuals with diabetes [49], weekly web usage decreased from 189 of 270 (70%) participants logging on during the first 6 weeks to 127 of 270 (47%) participants logging on during weeks 7-16.

Despite short-term attrition being noted as problematic, greater website engagement was generally associated with better behavioral and clinical outcomes. For example, more actively engaged individuals with diabetes showed greater evidence of disease management activity. McMahon and colleagues [55] noted that a larger number of website data uploads was associated with a larger decline in A1C (*P*=.019), while Nijland and colleagues [51] noted that highly active Web 2.0 users consumed medication more often than low/inactive users (*P*=.005) [51]. Richardson and colleagues [50] noted that online walking community participants who viewed more pages, or posted to the website more often, demonstrated larger increases in walking step counts (*P*<.001; *P*=.03). However, two RCTs studying individuals with type 2 diabetes suggested that self-monitoring using Web 2.0 did not improve medication adherence [49,58].

#### Functional Outcomes

Findings related to physical activity and nutrition outcomes were mixed. Three studies by Lorig and colleagues [56,59,60] reported conflicting results regarding the effect of Web 2.0 participation on physical activity. In RCTs of patients with musculoskeletal disorders [59] and type 2 diabetes [56], there were no improvements noted in self-reported aerobic, stretching, and strengthening exercise; whereas, a quasi-experimental study of Australians with one or more chronic conditions [60] noted improvements on weekly minutes of exercise and behavioral adherence. In other studies of individuals with diabetes [49,58], self-monitoring of physical activity behaviors improved with concomitant reductions in dietary fat intake.

#### Clinical Outcomes

For the most part, the Web 2.0 interventions tested did not meaningfully impact short-term clinical outcomes, although only 6 reviewed studies [47,49,55,57,58,61] measured the near-term effectiveness of biological and clinical outcomes. For example, over a 4-month study period, website engagement among individuals with diabetes was not associated with any improvements in biological or clinical outcomes [49].

### Adoption: Setting and Staff

At the setting level, five of the Web 2.0 interventions were operated by academic research centers [47,52,54,55,57], while four were administered by various health care foundations and clinics [49,51,53,58]. Only three reviewed studies [48,50,60] did not address issues of adoption at the setting level. Thirteen of the 15 reviewed studies (87%) discussed staff level characteristics associated with intervention adoption. Multidisciplinary teams of researchers and practitioners were actively involved in adopting the delivery of Web 2.0 interventions for individuals with chronic disease. Several studies of individuals with diabetes described team science initiatives and collaborations [49,51,55,57,58]. These Web 2.0 interventions were staffed by a variety of health professionals including (but not limited to): diabetes care managers [49,55,58], nutritionists [49,55,58], nurses [51,55,57], behavioral scientists [51], primary care physicians [51,58], pharmacists [55], psychologists [57], diabetes educators [55], and social workers [57]. In each of these studies, one health professional generally acted as the intervention gatekeeper by conducting an initial consultation with the participant. Following this preliminary consultation, routine online follow-up contacts were coordinated by a multidisciplinary set of providers. Often, these follow-up sessions or meetings specifically addressed the variety of concerns that a patient with diabetes is likely to encounter (eg, medication changes, depression, burnout, coping, healthy eating).

Other reviewed studies (not restricted to diabetes) also described how multidisciplinary researcher and practitioner teams came together to staff Web 2.0 chronic disease self-management interventions [47,52-54]. These research teams were responsible for developing and delivering online instructional units and managing program content and communications. In three of these studies [47,53,61], nurses took leading roles to execute patient-centered consultations, host weekly chat sessions and videoconferences, as well as schedule and coordinate follow-up sessions. Studies conducted by Lorig et al [56,59,60] reported the use of peer moderators (ie, individuals also living with a chronic condition who are trained to lead self-management training programs on the Internet) to staff online workshops and facilitate scheduled Web 2.0 intervention learning activities. On scales ranging from 0 to 4, the setting (mean=2.2, SD 1.26) and staff (mean=2.27, SD 1.58) level SQS scores were quite similar.

### Implementation: Program Delivery

Almost half (n=7) of the 15 reviewed studies did not provide a detailed evaluation of program costs (money, time, human resources expended), adaptations made to Web 2.0 interventions over time, or fidelity to the intervention protocol. The costs of intervention implementation (ie, money, time, human resource management) were addressed in only six (40%) of the reviewed studies [47,49,51,52,54,55]. Cost considerations included time spent training study staff [55], administration time operating the Web 2.0 intervention [49,54], and developmental costs creating web-based instructional materials [52]. Most studies discussing costs noted that Web 2.0 development and operation costs were high [47,52,54,55]; however, one reviewed study noted minimal financial and human resource burdens [51]. Adaptations were made to Web 2.0 interventions in only four reviewed studies [47,50,54,60]. Changes were necessary due to technical difficulties [47,60], increased staff needs [50], and requests for more scheduled web events to stimulate participant involvement and interaction [54]. Three of the reviewed studies [48,56,58] reported technical, usability, and integration challenges that even caused one study to stop early [58]. Less than half (n=6) of the reviewed studies [47,49,51,52,54,55] described formal process evaluations to assess program fidelity. Mean SQS scores on the Implementation subdimensions (ie, Web 2.0 uptake, intervention development, program integration) were not judged to be noteworthy (see Multimedia Appendix 4).

Multimedia Appendix 5 describes the Web 2.0 implementation characteristics for each retained study. The web design and user interface of all reviewed studies supported two main web architectures: (1) online discussion groups, forums, boards, and communities, and/or (2) individualized entry and upload of personal health data (eg, medication, blood glucose, weight, exercise frequency). Seven of the 15 reviewed studies (47%) described how patients uploaded their personal data to a web platform for review by a clinician or peer moderator [48,49,56,57,59-61]. Graphic displays of user performance meeting personal goals were tracked in five of these studies [49-51,58,61]. Asynchronous communication (ie, participants do not communicate concurrently with one another, sending/posting messages at different times) was used most often through email or an internal messaging system [47,49,51,55,56,58,61]. Several Web 2.0 interventions implemented a combination of asynchronous and synchronous (ie, direct communication where parties are present at the same time) communication features [47,48,51,54,55,61]. Participants reported discussion boards [52], resource pages [52,59], asynchronous electronic messaging [54], personal action plans [49], and individual progress reports [49] as being especially useful for interactive health communication.

Lorig et al [56,59,60] enabled participants to “self-tailor” their interactive learning experiences while participating in interventions. This empowering approach represents an innovative implementation strategy for Web 2.0 self-management [60]. Using this strategy, participants devise a periodic action plan for themselves according to what particular self-management activities (eg, use of cognitive symptom management techniques, drawing up fitness/exercise regimens, planning meals) they want to engage in over a set period of time. Then, they are asked to rate their self-efficacy for accomplishing these planned activities before participating in the tasks. This reflection encourages patients to think about doing what is “real” as opposed to what is “ideal” [60]. Self-tailoring operates under principles of self-efficacy theory [62] by supporting the participant to pursue mastery experiences over time to build self-confidence. Six other studies also alluded to implementing principles of self-tailoring by helping participants develop: (1) action plans, (2) “To Do” lists for attaining weekly goals, (3) symptom self-monitoring diaries, and (4) tailored reasons/strategies for goal attainment [49-51,54,56,58].

### Maintenance: Individual and Setting

At the individual level, there were mixed results on the effect of technical mishaps on patient exposure to Web 2.0 chronic disease self-management. In several studies [47,48,51], technical difficulties were associated with (1) decreased participant engagement, (2) lower intervention enrollment, and (3) increased nonusage attrition. Problems included lack of Internet access, unreliable wireless coverage, slowed performance due to proprietary security software, poor navigation structures, and overall trouble with log-ins. Several other studies did not report these types of long-term technical problems, however, and instead reported highly active participation for up to 1 year among participants [51,54,59-61]. Even comfort with using computers and the Internet improved among participants [52-54].

While only moderate 12-month improvements were noted in biological outcomes and self-reported health care utilization [56,59,60], there were other several notable long-term effects maintained at the individual level. Glasgow et al [58] and Lorig et al [60] found statistically significant improvements in health behaviors and health status. Several RCTs of individuals with diabetes reported that Web 2.0 participation was associated with improved generic health-related quality of life and a reduction in depressive symptoms [57], greater declines in A1C [55,56], and reductions in blood pressure [55,58].

At the setting level, 13 of the 15 reviewed studies (87%) did not describe any substantive measures taken to sustain Web 2.0 interventions past designated study time periods. The mean SQS scores on both the setting (mean=3.13, SD 2.26 on a scale from 0 to 7) and individual (mean=1.53, SD 1.25 on a scale from 0 to 3) levels of the Maintenance dimension were judged to be the lowest of all RE-AIM dimensions that were evaluated.

## Discussion

This review provides a synthesis of research studies that describe Web 2.0 chronic disease self-management inventions for older adults. *Healthy Aging 2.0* argues that the evolution of older e-patients using participatory Web 2.0 technologies (eg, social networking, telemedicine, mHealth applications) requires new methods for transforming current health care communications [63]. Several overarching recommendations gleaned from this literature review will be discussed in the context of RE-AIM to guide the planning, implementation, and evaluation of future chronic disease self-management Web 2.0 interventions.

### Reach

Some researchers have proposed that the “digital divide” in health promotion and disease management may be shrinking [24,64]. This systematic review indicated that the majority of reviewed interventions targeted only older adults with diabetes, and most involved small samples primarily consisting of white females in the United States. Additional research is needed among older adults with other types of chronic conditions (eg, arthritis, hypertension, COPD) to determine actual usage as well as disease-specific reasons for use and nonuse of Web 2.0 technologies. Understanding disease-specific factors is important, because the effects of Web 2.0 engagement will likely be stronger if health care practitioners determine the type of patients more likely to log in regularly as opposed to sporadically. Strengthening the breadth of Web 2.0 interventions to include multiple chronic conditions will likely have an adverse impact on reach however [58]. Therefore, we need to cost-effectively reach diverse samples of older adults who are managing a variety of comorbid conditions. More sufficiently powered studies should attempt to include underrepresented, medically underserved chronic disease patients to determine how these populations may benefit from Web 2.0 self-management support programs.

### Effectiveness

To date, researchers have insisted that too few high-quality Web-based interventions have been conducted to sufficiently test the effectiveness of different types of Internet-mediated interventions [20,65]. The majority of studies in this review (n=9), however, were theoretically based RCTs that provided a relatively high level of evidence. Older adults felt greater self-efficacy for managing their disease(s) and benefitted from interacting with health care providers and/or website moderators to receive feedback and support. When familiarity with Web 2.0 improves, older participants (especially those with low self-efficacy and social support) may gain knowledge, skills, and mastery experiences to reinforce recommended self-management strategies. Evidence suggests that greater Web 2.0 engagement may also be associated with more positive behavioral (ie, physical activity) and clinical (ie, HRQoL) outcomes; however, this review indicates that Web 2.0 self-management interventions have yet to meaningfully impact medication adherence, biological outcomes, and health care utilization among older adults.

In order for Web 2.0 self-management interventions to become core components of chronic disease management programs, more evidence is needed to support that Internet-mediated health ICTs are associated with improvements in health outcomes. For many of the reviewed studies, it was not clear which aspect or component of each intervention was most effective even though web log activity was monitored in almost all (93%) of the reviewed studies. As was indicated in our SQS quality assessment, impact evaluations assessing Web 2.0 engagement were generally lacking. This diminished the researchers’ ability to determine patient satisfaction with different ICT exposures and limited further insights into the primary usability problems leading to low usage. Future Web 2.0 studies should use impact evaluation frameworks to reveal the active components of multicomponent Web 2.0 interventions so that we may determine the contexts in which treatments are most effective [66] and also distinguish the right combination of human and computerized support necessary to facilitate sustained participation [58].

### Adoption

Among the studies that addressed adoption at the setting level, most described team science approaches to adopting Web 2.0 for chronic disease self-management support. Multidisciplinary groups of health care and ICT professionals built upon shared skills and experiences to develop chronic disease self-management interventions, primarily for individuals with diabetes. Given the increased emphasis on the coordination of chronic care teams [3], it is interesting to note the omnipresence of provider teams participating in the development of Web 2.0 interventions. The minimum administrative time burden associated with operating an interactive chronic disease self-management website may be quite high; thus, team-based approaches may reduce the administrative burden placed on individual health care units to operate Web 2.0 self-management support programs. Future studies should conduct more detailed setting and staff level analyses to determine whether operating Web 2.0 self-management interventions is feasible within existing public health and health care administration units.

### Implementation

Even though participants viewed Web 2.0 favorably, program implementation was not seamless. Most studies noted that Web 2.0 development and operation costs were high, and the majority of studies did not sufficiently evaluate implementation quality [67]. Less than half of the reviewed studies described formal process evaluations to assess program fidelity. Process evaluations are important to: (1) identify best-practice strategies for future programs, (2) reduce potential for technical difficulties, (3) determine the amount of time patients are willing to spend using Web 2.0 for self-management support, and (4) estimate the amount of human and financial resources necessary for high-quality delivery. Implementation costs include trained facilitators, online data collection/analysis, Web system testing and hosting, as well as back up services for technical anomalies [54]. Cost projections encourage program developers to consider issues of dosage, staff training/supervision procedures, and revising administrative and practice responsibilities of health care personnel. Different Web 2.0 models need to be evaluated economically before these resource-intensive interventions are disseminated to chronically ill older adults at the population level [47].

Asynchronous communication tools (ie, email, internal messaging systems, discussion boards) and personal tracking features (ie, graphical displays of uploaded data) were noted as some of the more useful interactive Web 2.0 components. Promising findings from multiple studies suggested that “self-tailored” Web 2.0 approaches may reduce health distress and activity limitation, improve health status, and foster patient engagement more so than less patient-centered Web 2.0 approaches. While these tools and strategies have shown promise in promoting interaction, it remains unclear how best to define and measure web engagement/participation among older participants [68]. Post hoc patient interviews in this population may be important for better understanding the engagement construct, especially since individual psychosocial characteristics may be highly associated with level of Internet use [49,58]. Some researchers have recommended that scatter plot displays of the relationship between engagement and outcomes be analyzed, along with logistic regression analyses that determine whether unique patient characteristics predict dichotomous threshold use indices for different Web 2.0 components [49,58]. Future research should determine engagement metrics that are important to evaluate during Web 2.0 chronic disease self-management interventions.

Integrating Web 2.0 self-management interventions into primary care settings seems like a logical next venue for implementation [58]. Patient-centered health care organizations can employ virtual communities to direct and support chronic disease patients [69]. Some research has shown that e-patients with chronic diseases want easy access to multiple interactive tools they can control and customize [70]. Other research indicates that patients prefer fewer system components that can be used repeatedly [71]. Flexible tools that give users greater control and choice may be more convenient and customizable, and thus result in greater patient satisfaction, sustained engagement, and more positive health outcomes. Forward-thinking implementation strategies should recognize patients as experts in their own disease process and management [54]. These types of progressive approaches are likely to generate on-line contexts that deliver more personalized self-management training experiences.

### Maintenance

Managing illness is a lifelong responsibility for chronically ill older adults, who often have to deal with physical limitations and increasingly difficult living conditions over time [72]. Regrettably, the reviewed studies were judged to be weakest on the Maintenance dimension of the RE-AIM evaluation framework. At 12 months, only moderate overall gains were observed in biological outcomes and health care utilization. There were, however, some long-term improvements to report in health behavior, health status, and even with respect to a few clinical markers [55,58-60]. Future studies should be designed to have longer follow-up periods to test whether positive 1-year intervention effects can be sustained among larger, more diverse samples of chronically ill patients over longer periods of time in spite of low-usage attrition or dropout [22].

Maintaining and expanding Web 2.0 for chronic disease self-management requires a better understanding of the barriers that prevent continuous access to the Internet. We do not yet fully understand which factors influence long-term use/nonuse of Web 2.0 because user attrition in older adult chronic disease populations is rarely examined in depth. Health care policy makers would be best served by accounting for the way older adults are using Web 2.0 technologies to research personal health choices and interact with health care experts [73]. Elements crucial to the maintenance of future interventions may include: (1) establishing multidisciplinary teams, (2) allowing adequate time for research and development, (3) securing sufficient resources for building and maintaining an online presence, and (4) developing an integrated process and impact evaluation framework [74]. Future interventions should continuously evaluate individual needs and system requirements to understand which intervention strategies are best suited for Web 2.0 [51].

It was interesting to note that as program exposure increased among participants, so too did comfort with using computers and the Internet. Future interventions should consider installing feedback mechanisms and triggers (eg, email reminders) that provide automated messages to motivate and inspire users to participate in interactive self-management experiences on the Internet. So-called “push factors” may influence persistent engagement and support longer-term use [75]. Currently, it is unclear which types of automated supports (eg, email alerts, text message reminders, inspiring testimonials) encourage more active involvement in Web 2.0 chronic disease self-management. Additional research should determine whether personalized feedback from a real person is more persuasive than computerized tailored feedback and how to achieve the most effective and cost-effective balance between automated and nonautomated correspondence when using Web 2.0 [51,58].

### Limitations

This study had several limitations. Although the search was comprehensive and systematic, using a rigorous method of searching and reviewing articles, some studies may have been overlooked due to lack of indexing in searched databases. Moreover, variable terminology used to describe web-based chronic disease self-management programs on the Internet could have led to missing certain eligible studies. As a result, the final sample of articles included in the study (n=15) may have been limited. In addition, several articles had relatively small sample sizes, which may have not been representative of the patient populations from which they were drawn. Consequently, our ability to generalize is limited. Diversity in the samples was also lacking. The samples in the reviewed studies consisted primarily of highly educated, white patients. Lower socioeconomic status populations, with low-literacy levels, were underrepresented in the reviewed studies.

The combination of dropouts and limited adherence to program activities may also have led to a misrepresentation of intervention effects. Participants who did participate in these interventions may have unintentionally (or intentionally) contributed dubious or outright false health information that may have negatively impacted other intervention participants. The lack of regulation when delivering self-management training opportunities via Web 2.0 may prompt false senses of empowerment to the extent that patients may even contest treatment options and decisions handed down from their health care providers.

### Conclusions

We can expect many specialized, patient-centered websites to arise in response to specific chronic disease information needs [76]. While Web 2.0 may help train chronically ill patients to make informed decisions and solve daily self-management problems [22,48], the effectiveness of Web 2.0 interventions for patients with chronic diseases remains a significant challenge [77]. There is concern that Web 2.0 tools are made available regardless of usability, acceptability, and/or associated outcomes [78]. To extend the reach of chronic disease self-management and promote more widespread Web 2.0 adoption across different health care settings and among multidisciplinary teams of health care providers, future research should attempt to determine how to create personally customizable content-sharing websites regarding healthy lifestyles, treatment options, and locating available health services. Given that older adults are the fastest growing group of novice computer users [79] and that the opportunity to reach these individuals will increase as older adults become “wired” for Internet access [6], researchers must actively explore how to improve the quality of these interventions for older populations.

A patient-centered, evidence-based framework is needed to design and deliver Web 2.0 technologies to older adults who may require specialized tools because of functional and cognitive impairments associated with aging [80]. Using results from this review in concert with the RE-AIM model may provide guidance for creating more patient-centered chronic disease self-management models that consider Web 2.0 user interfaces (technical, information architecture, aesthetic, and functional), communication features (synchronous or asynchronous), and learning modalities (low-literate instructional design). The effective translation of these strategies using Web 2.0 applications will likely result in new approaches for empowering, engaging, and educating older adults with chronic disease.

## Multimedia Appendix 1

SQS measurement criteria.

## Multimedia Appendix 2

SQS measurement items.

## Multimedia Appendix 3

RE-AIM Attributes of Reviewed Studies.

## Multimedia Appendix 4

SQS scores for articles.

## Multimedia Appendix 5

Web 2.0 Implementation Characteristics.

## Abbreviations

COPD: chronic obstructive pulmonary disease
HRQoL: health-related quality of life
ICT: information and communication technology
RCT: randomized controlled trial
RE-AIM: Reach, Efficacy, Adoption, Implementation, and Maintenance
SQS: study quality score

## References

[1] (2009). Centers for Disease Control and Prevention.

[2] World Health Organization.

[3] Centers for Disease Control and Prevention, AARP, American Medical Association (2009). Promoting Preventive Services for Adults 50-64: Community and Clinical Partnerships.

[4] Hesse BW, Hansen D, Finholt T, Munson S, Kellogg W, Thomas JC (2010). Social Participation in Health 2.0. Computer (Long Beach Calif).

[5] Fox S, Pucell K California Healthcare Foundation.

[6] Zickuhr K, Madden M Pew Research Center's Internet & American Life Project.

[7] Bennett GG, Glasgow RE (2009). The delivery of public health interventions via the Internet: actualizing their potential. Annu Rev Public Health.

[8] Clark NM (2003). Management of chronic disease by patients. Annu Rev Public Health.

[9] Griffiths F, Lindenmeyer A, Powell J, Lowe P, Thorogood M (2006). Why are health care interventions delivered over the internet? A systematic review of the published literature. J Med Internet Res.

[10] Madden M.

[11] Moeller P U.S. News and World Report site.

[12] Zickuhr K, Smith A Digital differences.

[13] Hawkins RP, Han JY, Pingree S, Shaw BR, Baker TB, Roberts LJ (2010). Interactivity and Presence of Three eHealth Interventions. Comput Human Behav.

[14] Capel S, Childs S, Banwell L, Heaford S (2007). Access to information and support for health: some potential issues and solutions for an ageing population. Health Informatics J.

[15] Murray E, Burns J, See TS, Lai R, Nazareth I (2005). Interactive Health Communication Applications for people with chronic disease. Cochrane Database Syst Rev.

[16] Pulman A (2010). A patient centred framework for improving LTC quality of life through Web 2.0 technology. Health Informatics J.

[17] Birnsteel L Web 2.0 in the Health Sector: Industry Review with UK Perspective Report.

[18] Car J, Sheikh A (2004). Email consultations in health care: 1--scope and effectiveness. BMJ.

[19] Podichetty V, Penn D (2004). The progressive roles of electronic medicine: benefits, concerns, and costs. Am J Med Sci.

[20] Samoocha D, Bruinvels DJ, Elbers NA, Anema JR, van der Beek AJ (2010). Effectiveness of web-based interventions on patient empowerment: a systematic review and meta-analysis. J Med Internet Res.

[21] Wantland DJ, Portillo CJ, Holzemer WL, Slaughter R, McGhee EM (2004). The effectiveness of Web-based vs. non-Web-based interventions: a meta-analysis of behavioral change outcomes. J Med Internet Res.

[22] Eland-de Kok P, van Os-Medendorp H, Vergouwe-Meijer A, Bruijnzeel-Koomen C, Ros W (2011). A systematic review of the effects of e-health on chronically ill patients. J Clin Nurs.

[23] Strecher V (2007). Internet methods for delivering behavioral and health-related interventions (eHealth). Annu Rev Clin Psychol.

[24] Jones S, Fox S (2009). Generations Online.

[25] de Clercq PA, Hasman A, Wolffenbuttel BH (2001). Design of a consumer health record for supporting the patient-centered management of chronic diseases. Stud Health Technol Inform.

[26] Hejlesen OK, Plougmann S, Ege BM, Larsen OV, Bek T, Cavan D (2001). Using the internet in patient-centred diabetes care for communication, education, and decision support. Stud Health Technol Inform.

[27] Loader BD, Muncer S, Burrows R, Pleace N, Nettleton S (2002). Medicine on the line? Computer-mediated social support and advice for people with diabetes. Int J Soc Welf.

[28] Laakso E, Armstrong K, Usher W (2012). Cyber-management of people with chronic disease: A potential solution to eHealth challenges. Health Educ J.

[29] Feldman MD (2000). Munchausen by Internet: detecting factitious illness and crisis on the Internet. South Med J.

[30] Russell C, Campbell A, Hughes I (2008). Ageing, social capital and the Internet: findings from an exploratory study of Australian 'silver surfers'. Australas J Ageing.

[31] Gustafson DH, Hawkins R, Boberg E, Pingree S, Serlin RE, Graziano F, Chan CL (1999). Impact of a patient-centered, computer-based health information/support system. Am J Prev Med.

[32] Overberg R, Otten W, de Man A, Toussaint P, Westenbrink J, Zwetsloot-Schonk B (2010). How breast cancer patients want to search for and retrieve information from stories of other patients on the internet: an online randomized controlled experiment. J Med Internet Res.

[33] McWilliam CL (2009). Patients, persons or partners? Involving those with chronic disease in their care. Chronic Illn.

[34] Center for Technology and Aging.

[35] Eysenbach G (2008). Medicine 2.0: social networking, collaboration, participation, apomediation, and openness. J Med Internet Res.

[36] Lorig KR, Holman H (2003). Self-management education: history, definition, outcomes, and mechanisms. Ann Behav Med.

[37] Newman S, Steed L, Mulligan K (2004). Self-management interventions for chronic illness. Lancet.

[38] O'Reilly T, Battelle J (2004). Opening Welcome. State of the Internet Industry Web 2.0 Conference.

[39] World Health Organization Definition of an Older or Elderly Person: Proposed Working Definition of an Older Person in Africa for the MDS Project.

[40] Glasgow RE, Vogt TM, Boles SM (1999). Evaluating the public health impact of health promotion interventions: the RE-AIM framework. Am J Public Health.

[41] Kessler RS, Purcell EP, Glasgow RE, Klesges LM, Benkeser RM, Peek CJ (2013). What Does It Mean to "Employ" the RE-AIM Model?. Eval Health Prof.

[42] Glasgow RE, Dickinson P, Fisher L, Christiansen S, Toobert DJ, Bender BG, Dickinson LM, Jortberg B, Estabrooks PA (2011). Use of RE-AIM to develop a multi-media facilitation tool for the patient-centered medical home. Implement Sci.

[43] Zaza S, Wright-De Agüero LK, Briss PA, Truman BI, Hopkins DP, Hennessy MH, Sosin DM, Anderson L, Carande-Kulis VG, Teutsch SM, Pappaioanou M (2000). Data collection instrument and procedure for systematic reviews in the Guide to Community Preventive Services. Task Force on Community Preventive Services. Am J Prev Med.

[44] Thomas BH, Ciliska D, Dobbins M, Micucci S (2004). A process for systematically reviewing the literature: providing the research evidence for public health nursing interventions. Worldviews Evid Based Nurs.

[45] Higgins JPT, Green S, The Cochrane Collaboration (2011). Cochrane Handbook for Systematic Reviews of Interventions. Version 5.1.0.

[46] Molleman GR, Ploeg MA, Hosman CM, Peters LH (2006). Preffi 2.0- a quality assessment tool. Promot Educ.

[47] Nguyen HQ, Donesky-Cuenco D, Wolpin S, Reinke LF, Benditt JO, Paul SM, Carrieri-Kohlman V (2008). Randomized controlled trial of an internet-based versus face-to-face dyspnea self-management program for patients with chronic obstructive pulmonary disease: pilot study. J Med Internet Res.

[48] Solomon M, Wagner SL, Goes J (2012). Effects of a Web-based intervention for adults with chronic conditions on patient activation: online randomized controlled trial. J Med Internet Res.

[49] Glasgow RE, Christiansen SM, Kurz D, King DK, Woolley T, Faber AJ, Estabrooks PA, Strycker L, Toobert D, Dickman J (2011). Engagement in a diabetes self-management website: usage patterns and generalizability of program use. J Med Internet Res.

[50] Richardson CR, Buis LR, Janney AW, Goodrich DE, Sen A, Hess ML, Mehari KS, Fortlage LA, Resnick PJ, Zikmund-Fisher BJ, Strecher VJ, Piette JD (2010). An online community improves adherence in an internet-mediated walking program. Part 1: results of a randomized controlled trial. J Med Internet Res.

[51] Nijland N, van Gemert-Pijnen JE, Kelders SM, Brandenburg BJ, Seydel ER (2011). Factors influencing the use of a Web-based application for supporting the self-care of patients with type 2 diabetes: a longitudinal study. J Med Internet Res.

[52] Cudney S, Weinert C (2012). An online approach to providing chronic illness self-management information. Comput Inform Nurs.

[53] Marziali E (2009). E-health program for patients with chronic disease. Telemed J E Health.

[54] Smarr KL, Musser DR, Shigaki CL, Johnson R, Hanson KD, Siva C (2011). Online self-management in rheumatoid arthritis: a patient-centered model application. Telemed J E Health.

[55] McMahon GT, Gomes HE, Hickson Hohne S, Hu TM, Levine BA, Conlin PR (2005). Web-based care management in patients with poorly controlled diabetes. Diabetes Care.

[56] Lorig K, Ritter PL, Laurent DD, Plant K, Green M, Jernigan VBB, Case S (2010). Online diabetes self-management program: a randomized study. Diabetes Care.

[57] Bond GE, Burr RL, Wolf FM, Feldt K (2010). The effects of a web-based intervention on psychosocial well-being among adults aged 60 and older with diabetes: a randomized trial. Diabetes Educ.

[58] Glasgow RE, Kurz D, King D, Dickman JM, Faber AJ, Halterman E, Woolley T, Toobert DJ, Strycker LA, Estabrooks PA, Osuna D, Ritzwoller D (2012). Twelve-month outcomes of an Internet-based diabetes self-management support program. Patient Educ Couns.

[59] Lorig KR, Ritter PL, Laurent DD, Plant K (2008). The internet-based arthritis self-management program: a one-year randomized trial for patients with arthritis or fibromyalgia. Arthritis Rheum.

[60] Lorig K, Ritter PL, Plant K, Laurent DD, Kelly P, Rowe S (2013). The South australia health chronic disease self-management internet trial. Health Educ Behav.

[61] Nguyen HQ, Donesky D, Reinke LF, Wolpin S, Chyall L, Benditt JO, Paul SM, Carrieri-Kohlman V (2012). Internet-Based Dyspnea Self-Management Support for Patients With Chronic Obstructive Pulmonary Disease. J Pain Symptom Manage.

[62] Bandura A (1997). Health Functioning. Self-efficacy: the exercise of control.

[63] Hall AK, Stellefson M, Bernhardt JM (2012). Healthy Aging 2.0: the potential of new media and technology. Prev Chronic Dis.

[64] Stellefson ML, Chaney EH, Chaney JD (2008). The digital divide in health education: Myth or reality?. Am J Health Educ.

[65] Webb TL, Joseph J, Yardley L, Michie S (2010). Using the internet to promote health behavior change: a systematic review and meta-analysis of the impact of theoretical basis, use of behavior change techniques, and mode of delivery on efficacy. J Med Internet Res.

[66] Eisenstein EL, Lobach DF, Montgomery P, Kawamoto K, Anstrom KJ (2007). Evaluating implementation fidelity in health information technology interventions. AMIA Annu Symp Proc.

[67] Borrelli B, Sepinwall D, Ernst D, Bellg AJ, Czajkowski S, Breger R, DeFrancesco C, Levesque C, Sharp DL, Ogedegbe G, Resnick B, Orwig D (2005). A new tool to assess treatment fidelity and evaluation of treatment fidelity across 10 years of health behavior research. J Consult Clin Psychol.

[68] Danaher BG, Seeley JR (2009). Methodological issues in research on web-based behavioral interventions. Ann Behav Med.

[69] Winkelman WJ, Choo CW (2003). Provider-sponsored virtual communities for chronic patients: improving health outcomes through organizational patient-centred knowledge management. Health Expect.

[70] Kerr C, Murray E, Stevenson F, Gore C, Nazareth I (2006). Internet interventions for long-term conditions: patient and caregiver quality criteria. J Med Internet Res.

[71] Hurling R, Catt M, Boni MD, Fairley BW, Hurst T, Murray P, Richardson A, Sodhi JS (2007). Using internet and mobile phone technology to deliver an automated physical activity program: randomized controlled trial. J Med Internet Res.

[72] Car J, Sheikh A (2004). Email consultations in health care: 2--acceptability and safe application. BMJ.

[73] Hardey M (2008). Public health and web 2.0. Perspect Public Health.

[74] Gold J, Pedrana AE, Stoove MA, Chang S, Howard S, Asselin J, Ilic O, Batrouney C, Hellard ME (2012). Developing health promotion interventions on social networking sites: recommendations from The FaceSpace Project. J Med Internet Res.

[75] Fogg B A behavior model for persuasive design.

[76] Ossebaard HC, Seydel ER, van Gemert-Pijnen L (2012). Online usability and patients with long-term conditions: a mixed-methods approach. Int J Med Inform.

[77] Kerr C, Murray E, Noble L, Morris R, Bottomley C, Stevenson F, Patterson D, Peacock R, Turner I, Jackson K, Nazareth I (2010). The potential of Web-based interventions for heart disease self-management: a mixed methods investigation. J Med Internet Res.

[78] Holt M Health 2.0 definition: Holt's definition.

[79] Fox S E-patients with a disability or chronic disease.

[80] Administration on Aging US Department of Health and Human Services.

