# Supplementary material for: Web 2.0 Chronic Disease Self-Management for Older Adults: A Systematic Review
Source: J Med Internet Res. 2013 Feb 14;15(2):e35. doi: 10.2196/jmir.2439 (PMC3636299; doi:10.2196/jmir.2439)
Supplement: Supplementary file 1 [file jmir_v15i2e35_app1.pdf]

Multimedia Appendix 1. SQS measurement criteria.

RE-AIM Dimension	Quality criteria	Criteria Measure (min-max score)	
1. Reach	1a. Representativeness of sample	1a.1. Sampling frame	(0-1)
		1a.2. Screening criteria	(0-1)
		1a.3. Response rate	(0-1)
	Subscale Range:	(0-3)	
2. Efficacy	2a. Suitability of study design	2a.1. Power calculation	(0-1)
		2a.2. Level of evidence	(1-5)
		2a.3. Comparison groups	(0-1)
	Subscale Range:	(1-7)	
	2b. Credibility of data collection	2b.1. Missing data procedure	(0-1)
		2b.2. Evidence of reliability and validity	(0-1)
		2b.3. Location of measurement	(0-1)
	Subscale Range:	(0-3)	
	2c. Evaluation	2c.1. Clarity of evaluation principles	(0-2)
		2c.2. Theoretical rationale	(0-2)
		2c.3. Process	(0-2)
		2c.4. Effect	(0-2)
		2c.5. Type of change	(0-2)
		2c.6. Changes attributable to intervention	(0-2)
	Subscale Range:	(0-12)	
	2d. Statistical Analysis	2d.1. Suitability of analysis	(0-1)
		2d.2. P-values reporting	(0-1)
		2d.3. Effect size reporting	(0-1)

RE-AIM Dimension	Quality criteria	Criteria Measure (min-max score)	
	Subscale Range:	(0-3)	
3. Adoption	3a. Setting level	3a.1. Feasibility	(0-2)
		3a.2. Incorporation into existing structure	(0-2)
	Subscale Range:	(0-4)	
	3b. Staff level	3b.1. Expertise of project managers(s)	(0-2)
		3b.2. Stakeholder feedback	(0-2)
	Subscale Range:	(0-4)	
4. Implementation	4a. Web 2.0 Uptake	4a.1. Accessibility	(0-1)
		4a.2. Participant adherence	(0-1)
		4a.3. Duration (dosage) and intensity	(0-2)
	Subscale Range:	(0-4)	
	4b. Intervention development	4b.1. Incentives for Participation	(0-1)
		4b.2. Fitting strategies and methods to user	(0-2)
	Subscale Range:	(0-3)	
	4c. Program Integration	4c.1. Effectiveness of implementation techniques	(0-2)
		4c.2. Intervention coherence	(0-2)
		4c.3. Pretest	(0-2)
		4c.4. Monitoring and gathering feedback	(0-2)
	Subscale Range:	(0-8)	
5. Maintenance	5a. Setting level	5a.1. Support/Commitment for Program Maintenance	(0-2)
		5a.2. Capacity to maintain program	(0-2)
		5a.3. Leadership to maintain program	(0-2)

RE-AIM Dimension	Quality criteria	Criteria Measure (min-max score)	
		5a.4. Policy development	(0-1)
	Subscale Range:	(0-7)	
	5b. Individual level	5b.1. Positive effects observed ≥ 6 months	(0-1)
		5b.2. Positive effects observed ≥ 1 year	(0-1)
		5b.3. Long-term attrition ≤ 30%	(0-1)
	Subscale Range:	(0-3)	
		Total SQS Score:	(0-61)
