# Supplementary material for: Web 2.0 Chronic Disease Self-Management for Older Adults: A Systematic Review
Source: J Med Internet Res. 2013 Feb 14;15(2):e35. doi: 10.2196/jmir.2439 (PMC3636299; doi:10.2196/jmir.2439)
Supplement: Supplementary file 2 [file jmir_v15i2e35_app2.pdf]

Multimedia Appendix 2. SQS measurement items.

RE-AIM Dimension	Criteria Measure	Item	Response(s)
1. Reach	1a.1. Sampling frame [43]	Did the author(s) specify the sampling frame or methods of sample selection in the study population?	0 = No; 1 = Yes
	1a.2. Screening criteria [43]	Did author(s) specify the screening criteria for study eligibility?	0 = No; 1 = Yes
	1a.3. Response rate [44]	Were the study samples randomly recruited from the population with a response rate of at least 60%?	0 = No; 1 = Yes
2. Efficacy	2a.1. Power calculation [43]	Was a power calculation conducted?	0 = No; 1 = Yes
	2a.2. Level of evidence [45]	Indicate the level of evidence for the study design	5 = Experimental;<br>4 = Quasi-experimental studies;<br>3 = Controlled observational;<br>3 = Cohort;<br>3 = Case control;<br>2 = Observational studies without control;<br>1 = Expert opinion based on theory, laboratory research or consensus
	2a.3. Comparison groups [44]	Were baseline characteristics of the comparison groups comparable OR if there were important differences in potential confounders were these appropriately adjusted for in the analysis?	0 = No; 1 = Yes
	2b.1. Missing data procedure (New item)	Were missing data handling appropriately (ie procedures of how missing data were handled was described)?	0 = No; 1 = Yes
	2b.2. Evidence of reliability and validity [44]	Were the data tools used shown to be credible (eg shown to be valid and reliable in published research, OR in a pilot study, OR taken from a published national survey, OR recognized as acceptable measure)?	0 = No; 1 = Yes
	2b.3. Location of measurement [43]	Where were outcomes measured?	0 = different setting from intervention setting; 1 = same as intervention setting
	2c.1. Clarity of evaluation principles [Evaluation subscale – 46]	Clarity and agreement on principles of evaluation are rated:	0 = weak;<br>1 = moderate;<br>2 = strong

RE-AIM Dimension	Criteria Measure	Item	Response(s)
	2c.2. Theoretical rationale [Intervention Development subscale – 46]	Theory used and described (assess strength):	0 = weak;<br>0 = not reported;<br>1 = moderate;<br>2 = strong
	2c.3. Process [Evaluation subscale – 46]	Process evaluation is rated:	0 = weak;<br>1 = moderate;<br>2 = strong
	2c.4. Effect [Evaluation subscale – 46]	Effect evaluation is rated:	0 = weak;<br>1 = moderate;<br>2 = strong
	2c.5. Type of change [Evaluation subscale – 46]	What type of change has been measured?	0 = weak;<br>1 = moderate;<br>2 = strong
	2c.6. Changes attributable to intervention [Evaluation subscale – 46]	What is the strength of the assessment of if the intervention caused the change(s) reported?	0 = weak;<br>1 = moderate;<br>2 = strong
	2d.1. Suitability of analysis (New item)	Were the statistical analyses used suitable to answer the research question(s) posed?	0 = No; 1 = Yes
	2d.2. P-values reporting [43]	Were p-values were given for outcome measures?	0 = No; 1 = Yes
	2d.3. Effect size reporting [43]	Were effect sizes reported for outcome measures?	0 = No; 1 = Yes
3. Adoption	3a.1. Feasibility [Implementation subscale – 46]	Feasibility of program in existing practice (assess strength):	0 = weak;<br>0 = not reported;<br>1 = moderate;<br>2 = strong
	3a.2. Incorporation into existing structure [Implementation subscale – 46]	Incorporation into existing structure (assess strength):	0 = weak;<br>0 = not reported;<br>1 = moderate;<br>2 = strong
	3b.1. Expertise and characteristics of project manager(s) [Contextual Conditions and Feasibility subscale – 46]	Expertise and characteristics of project manager (assess strength):	0 = weak;<br>0 = not reported;<br>1 = moderate;<br>2 = strong
	3b.2. Stakeholder feedback [Evaluation subscale – 46]	How would you rate the feedback given to stakeholders?	0 = weak;<br>1 = moderate;<br>2 = strong
4. Implementation	4a.1. Accessibility (New item)	Was the web location easy to access for subjects?	0 = No; 1 = Yes
	4a.2. Participant adherence [44]	Were outcomes studied in a panel of respondents with a short -term attrition rate of less than 30% OR were results based on a cross-sectional design with at least 200 participants included in analysis in each wave?	0 = No; 1 = Yes

RE-AIM Dimension	Criteria Measure	Item	Response(s)
	4a.3. Duration (dosage) and intensity of intervention exposure [Intervention development subscale – 46]	Duration and intensity (assess strength):	0 = weak;<br>1 = moderate;<br>2 = strong
	4b.1. Incentives for Participation (New Item)	Were incentives given for program participation?	0 = No; 1 = Yes
	4b.2. Fitting strategies and methods to user culture [Intervention Development subscale – 46]	Fitting of program to "culture" (assess strength):	0 = weak;<br>0 = not reported;<br>1 = moderate;<br>2 = strong
	4c.1. Effectiveness of implementation techniques [Implementation subscale – 46]	Effectiveness of implementation techniques (including: room for personalized approach, feedback on effects, use of reward strategies, removing barriers to preferred behavior, mobilizing social support, training skills, arranging follow-up, goal setting, and interactive approach):	0 = weak;<br>0 = not reported;<br>1 = moderate;<br>2 = strong
	4c.2. Intervention coherence [Implementation subscale – 46]	Coherence of interventions (assess strength):	0 = weak;<br>0 = not reported;<br>1 = moderate;<br>2 = strong
	4c.3. Pretest [Implementation subscale – 46]	Pretest (assess strength):	0 = weak;<br>0 = not reported;<br>1 = moderate;<br>2 = strong
	4c.4. Monitoring and gathering feedback [Implementation subscale – 46]	Monitoring and generating feedback (assess strength):	0 = weak;<br>0 = not reported;<br>1 = moderate;<br>2 = strong
5. Maintenance	5a.1. Support/Commitment for Program Maintenance [Contextual Conditions and Feasibility subscale – 46]	Support/Commitment for Maintenance of program (assess strength):	0 = weak;<br>0 = not reported;<br>1 = moderate;<br>2 = strong
	5a.2. Capacity to maintain program [Contextual Conditions and Feasibility subscale – 46]	Capacity to maintain program (assess strength):	0 = weak;<br>0 = not reported;<br>1 = moderate;<br>2 = strong
	5a.3. Leadership to maintain program [Contextual Conditions and Feasibility subscale – 46]	Leadership to maintain program (assess strength):	0 = weak;<br>0 = not reported;<br>1 = moderate;<br>2 = strong
	5a.4. Policy development (New Item)	Was a policy developed to assist in maintenance of program?	0 = No; 1 = Yes
	5b.1. Individual level effects ≥ 6 months [41]	Were broad outcomes observed ≥ 6 months follow-up after treatment?	0 = No; 1 = Yes

RE-AIM Dimension	Criteria Measure	Item	Response(s)
	5b.2. Individual level effects ≥ 1 year [41]	Were broad outcomes observed ≥ 1 year follow-up after treatment?	0 = No; 1 = Yes
	5b.3. Long-term attrition ≤ 30% [41]	Were long-term outcomes studied in a panel of respondents with an attrition rate of less than 30%?	0 = No; 1 = Yes
