# Supplementary material for: Web 2.0 Chronic Disease Self-Management for Older Adults: A Systematic Review
Source: J Med Internet Res. 2013 Feb 14;15(2):e35. doi: 10.2196/jmir.2439 (PMC3636299; doi:10.2196/jmir.2439)
Supplement: Supplementary file 3 [file jmir_v15i2e35_app3.pdf]

Author(s); Year	Purpose	Sample Size (n)	Reach	Effectiveness	Adoption	Implementation	Maintenance
Nguyen HQ, Donesky-Cuenco D, Wolpin S, Reinke LF, Benditt JO, Paul SM, Carrieri-Kohlman V; 2008 [47]	Determine the effect of Internet-based dyspnea self-management program (eDSMP) compared to a face-to-face dyspnea self-management program (fDSMP) in people living with COPD.	n=39	Representativeness: Heterogeneous group of adults with COPD; Mean Age (SD): 69 (8.5)<br>Participation Rate: 17 of 39 participants (44%) female; 38 of 39 participants (97%) are White<br>Country: USA	Design: RCT<br>Theoretical Framework: Social cognitive, self-management, and pathophysiological theories<br>Major Findings: Participants in both programs showed similar clinically meaningful changes in dyspnea with ADL from baseline to 3 months (fDSMP: + 3.3 points; eDSMP: + 3.5 points) (no statistically significant differences between groups). Self-reported endurance exercise time (P = .001), physical functioning (P=.04), and self-efficacy for managing dyspnea (P = .02) improved over time in both groups. Decreased participant engagement with the Web and PDA tools during the intervention, which was reflected in the number of Web log-ins and exercise and symptom entries via the website and/or PDA.	Setting: Conducted at two academic medical centers<br>Staff: A study nurse conducted the baseline assessments, intervention consultations, weekly chat sessions, and follow-ups. Investigators not directly involved in the intervention conducted semi-structured interviews at final study visit.	Cost: Project noted to be resource-intensive<br>Intervention Adaptation: Numerous technical problems noted.<br>Process Fidelity: Significant technical and usability challenges caused study to stop early.	Individual: Improvements in dyspnea with ADL were sustained at 6 months in both groups, but more so for fDSMP (fDSMP: + 4.0 points; eDSMP: + 2.5 points).<br>Setting: Not addressed.
Solomon M, Wagner S, Goes J; 2012 [48]	Explore the effect of a Web-based intervention on the patient activation (i.e., attitudes toward knowledge, skills, and confidence in self-management) of patients with chronic health conditions.	n=201	Representativeness: Asthma, hypertension or diabetes<br>Participation Rate: 99 of 201 participants (49%) aged 55-64; 104 of 201 participants (52%) female; 173 of 201 participants (86%) White<br>Country: USA	Design: RCT<br>Theoretical Framework: N/A<br>Major Findings: When controlling for baseline patient activation scores, a positive yet small effect on the patient activation level of participants was observed in the Web-based intervention group when compared to similar control group [$F(1,123) = 4.438$, $P = .04$, $r^2 = .04$)]. Patients in the intervention group with more advanced patient activation (stage 4) did not demonstrate significant changes when compared with participants beginning at the first three stages of patient activation.	Setting: Not addressed.<br>Staff: Not addressed.	Cost: Not addressed.<br>Intervention Adaptation: None identified.<br>Process Fidelity: Not addressed.	Individual: The intervention group had 41 of 101 participants (41%) drop out of the study versus having only 32 of 100 participants (32%) leaving the study in the control group.<br>Setting: Not addressed.
Glasgow RE, Christiansen SM, Kurz D, Woolley T, Faber AJ, Estabrooks PA, Strycker L, Toobert D, Dickman J; 2011 [49]	Report the (1) overall rate of use of the My Path/Mi Camino diabetes self-management website; (2) components of website that were used most/least often; (3) participant characteristics associated with web engagement; & (4) associations between 4-month engagement and medication adherence, exercise and food choices.	n=270	Representativeness: Heterogeneous group of adults with type 2 diabetes; mean age (SD) = 58 (9.3)<br>Participation Rate: 130 of 270 participants (48%) female; 181 of 270 participants (67%) White<br>Country: USA	Design: RCT<br>Theoretical Framework: Social ecological theory & the 5 As self-management model<br>Major Findings: Over 4 months, website engagement was not associated with any biological or clinical outcomes or medication adherence, yet was related to healthy eating (r = .29; *P* = .04) reduction of dietary fat (r = -.31; *P* = .001), and exercise improvement (r = .20; *P* = .033). Website sections “Track My Progress”, “Action Plan”, and “My ABCs” were the 3 most used sections of the site (in order of use).	Setting: Conducted in five primary care clinics within Kaiser Permanente Colorado.<br>Staff: Research staff conducted an initial visit and made follow-up calls. A diabetes care manager completed a second call to the social support group. A nutritionist held a healthy eating group session within the social support group.	Cost: Patients spent an average of 7 minutes per visit on the website.<br>Intervention Adaptation: None identified.<br>Process Fidelity: 75% of participants entered self-monitoring data at least once per week.	Individual: Website usage decreased from 189 of 270 (70%) of participants visiting weekly during the first 6 weeks to 127 of 270 (47%) during weeks 7-16.<br>Setting: Not addressed.

Author(s); Year	Purpose	Sample Size (n)	Reach	Effectiveness	Adoption	Implementation	Maintenance
Richardson CR, Buis LR, Janney AW, Goodrich DE, Sen A, Hess ML, Mehari KS, Fortalge LA, Resnick PJ, Zikmund-Fisher BJ, Strecher VJ, Piette JD; 2010 [50]	Measure the impact of adding online community features to an Internet-mediated walking program on participant attrition and average daily step counts.	n=324	Representativeness:: Overweight, Type 2 Diabetes, or coronary artery disease Mean Age (SD): 52 (11.4) Participation Rate: 210 of 324 participants (65%) female; 279 of 324 participants (86%) are White Country: USA	Design: RCT Theoretical Framework: Social-cognitive theory and social influence theories Major Findings: Both arms of the intervention significantly increased average daily steps (no differences between treatment arms, *P* = .82). Online community participants with more pages viewed or more posts had larger increases in daily step counts and greater self-reported motivation.	Setting: Not addressed. Staff: Not addressed.	Cost: Not addressed. Intervention Adaptation: To stimulate online community involvement, significant research staff contributions to online community was required. Process Fidelity: Not addressed.	Individual: Participants in both arms who reported having social support at the end of the study were more likely to increase their step counts (*P* = .01). Setting: Participants received a 1-year free subscription to commercial Internet-mediated walking program.
Nijland N, van Gemert-Pijnen J, Kelders SM, Brandenburg BJ, Seydel ER; 2011 [51]	Explore factors that influence the initial and long-term use of a Web-based application (DiabetesCoach) for supporting self-care of patients with type 2 diabetes.	n=50	Representativeness: Type II Diabetes; Mean Age (SD): 61 (N/A); Participation Rate: 50 of 350 invited patients enrolled (14%); 13 of 50 participants (26%) female; 47 of 50 (93%) Dutch origin Country: The Netherlands	Design: Cross-sectional (convenience Representativeness) Theoretical Framework: Grounded theory Major Findings: DiabetesCoach was predominantly used for interactive features such as online monitoring, personal data entry, and nurse email contacts. Patients felt more closely monitored by their nurse and were encouraged to play a more active role in self-management. Highly active users were significantly more often medication users than low/inactive users (*P* = .005) and had a longer diabetes duration (*P* = .03).	Setting: Three out of 10 primary health care practices in the primary health care foundation in the Netherlands volunteered to take part in the DiabetesCoach project. Staff: DiabetesCoach was developed by Medicinfo in collaboration with general practitioners, nurses, patients, behavioral scientists and vendors. Nurses reviewed participant data.	Cost: Developmental and operational costs associated with the application were relatively low. Intervention Adaptation: None identified. Process Fidelity: Not addressed.	Individual: Only 16 of 50 (32%) enrollees did not continue using the web application for the full duration of the study period; 34 of 50 (68%) enrollees were continuous users, with 16 of 50 (32%) of these enrollees defined as highly active users. Setting: Barriers to long-term use included absence of "push" factors or reminders to log-in on the website.
Cudney S, Weinert C; 2012 [52]	Describe an online approach to providing chronic illness self-management information to rural women with chronic illness.	n=309	Representativeness: Rural women with at least one chronic disease; mean age (SD) = 56 years (N/A) Participation Rate: 287 of 309 participants (93%) are White Country: USA	Design: RCT Theoretical Framework: N/A Major Findings: Perceptions of computer skills and comfort level using the computer increased significantly for the intervention group, with no associated significant increases within the control group (computer skills mean = 6.23 to 6.84; *P* = .000; comfort level mean = 7.33 to 7.71; *P* = .007).	Setting: WebCT licensed by academic institutions was used to deliver intervention. Staff: Research team (which consisted of experts in specific areas of self-care and familiar with self-management literature) developed the online health teaching units	Cost: Designing quality health teaching units was noted to be a time-consuming task of the research team. Intervention Adaptation: None identified. Process Fidelity: Not addressed.	Individual: Resource utilization (such as helpful Internet links and discussion forums) was rated as the most positive intervention attribute during the study time period (mean = 4.84, SD not reported). Setting: Not addressed.
Marziali E; 2009 [53]	Examine the feasibility and benefits of an Internet-based videoconferencing healthcare support program for community-dwelling older adults with chronic disease.	n=18	Representativeness: One or more chronic conditions; Mean Age (SD): 61(N/A) Participation Rate: 15 of 18 participants (83%) female; race not given Country: Canada	Design: Qualitative (semi-structured interviews) Theoretical Framework: N/A Major Findings: Participants responded positively to using technology to communicate with health professionals and group members and reported reductions in feelings of loneliness and isolation. More than half of participants never used computers; yet, 14 of 18 participants (78%) indicated the website was easy to use	Setting: The project was located in a community health clinic in a large urban center. Staff: First ten weekly videoconferences were led by a professional healthcare provider.	Cost: Not addressed. Intervention Adaptation: None identified. Process Fidelity: Not addressed.	Individual: 17 of 18 participants (95%) felt that using computers to meet for group was moderately useful to very positive. Setting: Not addressed.

Author(s); Year	Purpose	Sample Size (n)	Reach	Effectiveness	Adoption	Implementation	Maintenance
Smarr KL, Musser DR, Shigaki CL, Johnson R, Hanson KD, Siva C; 2011 [54]	Describe the online transformation of an empirically validated, clinic-based self-management program for RA.	n=33	Representativeness:: Rheumatoid Arthritis (RA); Mean Age (SD): N/A<br>Participation Rate: gender: not reported; race: not reported<br>Country: USA	Design: Convenience Representativeness<br>Theoretical Framework: Cognitive-behavioral approaches to enhance self-efficacy, positive coping, and proactive behavior change<br>Major Findings: Participants are eager to receive this type of home-based programming and readily engaged program with few technical difficulties. “Message” feature was the most popular interactive feature	Setting: The online self-management website, RAHelp.org, was a project run through the Missouri Arthritis and Rehabilitation Research and Training Center.<br>Staff: Clinicians and arthritis professionals regularly monitored individual and community activity and managed the use of program content.	Cost: Estimated minimum administrative burden associated with operating online program was estimated at 7 h per member.<br>Intervention Adaptation: Because of the limited success of impromptu chats, biweekly scheduled events were initiated to provide more opportunities for substantive group chat.<br>Process Fidelity: Few difficulties navigating the technical environment. Chat feature was the least utilized likely because of users being in different time zones.	Individual: Not addressed.<br>Setting: Not addressed.
McMahon GT, Gomes HE, Hohne SH, Hu TM, Levine BA, Conlin PR; 2005 [55]	Assess the effects of web-based care management on glucose and blood pressure control over 12 months in patients with poorly controlled diabetes.	n=104	Representativeness: Diabetes with levels of HbA1c (A1C) ≥ 9%; Mean Age (SD): 64 (7)<br>Participation Rate: 103 of 104 participants (99%) male; race not given<br>Country: USA	Design: RCT<br>Theoretical Framework: N/A<br>Major Findings: There was a greater decline in A1C over time in the web-based group when compared with usual care group (*P* =. 048). A larger number of website data uploads was associated with a larger decline in A1C (*P* =. 019).	Setting: The study was conducted at the Department of Veterans Affairs Boston Healthcare system.<br>Staff: Participants met with a nurse, nutritionist, and pharmacist who were all certified diabetes educators. The MyCareTeam website was designed and hosted at the Imaging Science and Information Systems Center at Georgetown University Medical Center. An advanced practice nurse and diabetes educator reviewed participant data and provided recommendations. A care manager would suggest medication changes and the primary care provider would enter medication changes directly into the pharmacy’s electronic ordering system. Computer training and support was provided by the study staff.	Cost: Computer training and support provided by study staff average 2.3h per subject.<br>Intervention Adaptation: None identified.<br>Process Fidelity: Not addressed.	Individual: Over 12 months, systolic blood pressure (*P* = .009) and cholesterol (*P* = .047) also improved in the web-based group.<br>Setting: Not addressed.
Lorig K, Ritter PL, Laurent DD, Plant K, Green M, Blue Bird Jernigan V, Case Siobhan; 2010 [56]	Evaluate the effectiveness of an online diabetes self-management program including American Indians/Alaskan Natives (AI/AN) participants.	n=761	Representativeness: Heterogeneous group of adults with type 2 diabetes; mean age (SD): 54.3 (9.9);<br>Participation Rate: 73%, female, 76% White, 110 of 761 (14.5%) were American Indians/Alaskan Natives<br>Country: USA	Design: RCT<br>Theoretical Framework: Self-efficacy theory<br>Major Findings: There were few changes in other health or behavioral indicators following intervention. The AI/AN program participants demonstrated improvements in health distress and activity limitation compared with usual-care control subjects.	Setting: Password protected website access.<br>Staff: Peer moderators lead online workshops.	Cost: Not addressed.<br>Intervention Adaptation: None identified.<br>Process Fidelity: A1c was not measured at 18 months due to problems with laboratory.<br>.	Individual: At 6 months, A1C, patient activation, and self-efficacy were improved for participants in online program compared with usual care control subjects (*P*<0.05). At 18 months, self-efficacy (*P*= .007) and patient activation (*P*= .016) were improved for program participants.<br>Setting: Not addressed.

Author(s); Year	Purpose	Sample Size (n)	Reach	Effectiveness	Adoption	Implementation	Maintenance
Bond GE, Burr RL, Wolf FM, Feldt K; 2010 [57]	Investigate the impact of a 6-month Web-based intervention on the psychosocial well-being of older adults with diabetes.	n=62	Representativeness: Diagnosis of diabetes for at least 1 year; Mean Age (SD) = 67 years (5.9 years)<br>Participation Rate: 28 of 62 participants (45%) female, 54 of 62 participants (87%) are White<br>Country: USA	Design: RCT<br>Theoretical Framework: Self-efficacy theory<br>Major Findings: Web-based intervention group showed statistically significant improvements, on the measures of depression, social support, quality of life, and self-efficacy, when controlling for all baseline measures [*F* (4, 48) = 4.03; *P* = .007].	Setting: Subjected enrolled through the University of Washington Diabetes Center, Puget Sound Health System<br>Staff: A study nurse monitored participants' logs and communicated with them via e-mail and chat. The principal investigator held a formal weekly discussion group using MSN Messenger software. The group was related to depression, burnout, and coping, and were led by either an MSN social worker or a PhD psychologist.	Cost: Not addressed.<br>Intervention Adaptation: None identified.<br>Process Fidelity: Not addressed.	Individual: Not addressed.<br>Setting: Not addressed.
Glasgow RE, Kurz D, King D, Dickman JM, Faber AJ, Halterman E, Woolley T, Toobert DJ, Strycker LA, Estabrooks PA, Osuna D, Ritzwoller D; 2012 [58]	Determine the effects of an internet-based diabetes self-management program, with (CASM+) and without (CASM) additional telephone support, and as compared to enhanced usual care (EUC).	n=463	Representativeness: Heterogeneous group of adults with type 2 diabetes; mean age (SD): 58 (9.2);<br>Participation Rate: 231 of 463 participants (50%) female, 333 of 463 participants (72%) are White<br>Country: USA	Design: RCT<br>Theoretical Framework: Social ecological theory & the 5 As self-management model<br>Major Findings: Participants in the CASM/CASM+ conditions improved significantly more than EUC over 12 months (Cohen's *d* ES = .09 to .16) in eating habits, fat intake, and physical activity. CASM+ condition did not improve significantly more than CASM in any analysis. Latino ethnicity was significant moderator of change in blood pressure (*P* = .006).	Setting: The self-administered, computer-assisted study was conducted in five primary care clinics within Kaiser Permanente Colorado.<br>Staff: Follow-up calls were made by a diabetes care coordinator. Group sessions were led by a nutritionist and a family physician.	Cost: Not addressed.<br>Intervention Adaptation: None identified.<br>Process Fidelity: The program was not highly integrated with the patient's primary health care.	Individual: Long-term attrition was 73 of 231 participants (31.4%) in the CASM group and 59 of 232 (25.3%) in the CASM+ group. Attrition was significantly higher for CASM+ than in the EUC condition. Visits to the website declined considerably from an average of almost 11 times per month in the initial month to an average of fewer than three times per month in month 12 (no differences between CASM and CASM+ conditions).<br>Setting: Not addressed.
Lorig KR, Ritter PL, Laurent DD, Plant K; 2008 [59]	Determine the efficacy of an Internet-based Arthritis Self-Management Program (ASMP) as a resource for arthritis patients unable or unwilling to attend small-group ASMPs.	n=855	Representativeness:: Rheumatoid arthritis (RA), Osteoarthritis (OA), or Fibromyalgia; Mean Age (SD): 52 (N/A)<br>Participation Rate: 770 of 855 participants (90%) female, 787 of 855 participants (92%) are White<br>Country: USA	Design: RCT<br>Theoretical Framework: Self-efficacy theory<br>Major Findings: At 6 months, none of the health behaviors or health utilization outcome measures had statistically significant time-randomization interactions. Participants logged in a mean of 31.6 (SD=24.5) times over 6 weeks. Each workshop of 25 participants generated between 400 and 600 posts to The Discussion Center.	Setting: Password protected website complemented with interaction.<br>Staff: Peer moderators lead online workshops.	Cost: Not addressed.<br>Intervention Adaptation: None identified.<br>Process Fidelity: Not addressed.	Individual: At 1 year, *health distress* (*P* < .001), *activity limitation* (*P* < .001), *self-reported global health* (*P* = .004), *pain* (*P* < .001), and *self-efficacy* (*P*= .003) had significant time-randomization interactions. Participants reported that they found The Learning Center and Discussion Center very helpful during the intervention time period.<br>Setting: Not addressed.

Author(s); Year	Purpose	Sample Size (n)	Reach	Effectiveness	Adoption	Implementation	Maintenance
Lorig K, Ritter PL, Plant K, Laurent DD, Kelly P, Rowe S; 2012 [60]	Evaluate the effectiveness of an online chronic disease self-management program for residents of South Australia.	n=254	Representativeness: One or more chronic conditions; 113 of 254 participants (44.5%) ≥ 56 years of age<br>Participation Rate: 173 of 254 participants (68%) female; 250 of 254 participants (98%) White; varied from 246 of 254 (97%) for Session 1 to 165 of 254 (65%) for Session 6.<br>Country: Australia	Design: Quasi-experimental<br>Theoretical Framework: Self-efficacy theory<br>Major Findings: Significant improvements (*P* = .047) were found at 6 months for three health status measures, four health behaviors, and visits to the ER even after Bonferroni correction for multiple comparisons.	Setting: Not addressed.<br>Staff: Peer moderators lead online workshops and facilitated the intervention process.	Cost: Not addressed.<br>Intervention Adaptation: As the result of a programming problem, 58 participants in three workshops were not sent the automated e-mail asking them to complete the 6-month questionnaire. This resulted in a reduced number of cases and some reduction in statistical power.<br>Process Fidelity: The mean number of logons per participant was 25.4 times (SD=1.7) over 12 months. The mean number of sessions participated in was 4.8 per week, out of six possible weekly sessions.	Individual: At 12 months, two health status indicators (illness intrusiveness role function, health distress), five health behaviors (communication with doctor, minutes of exercise in past week, minutes of strength/stretch exercise, adherence, and self-efficacy to manage conditions), and ER visits were still improved.<br>Setting: Not addressed.
Nguyen HQ, Donesky D, Reinke LF, Wolpin S, Chyall L, Benditt JO, Paul SM, Carrieri-Kohlman V; 2012 [61]	Test the efficacy of an Internet-based and face-face dyspnea self-management program compared with a general health education control on the primary outcome of dyspnea with activities of daily living.	n=125	Representativeness: Heterogeneous group of adults with COPD; mean age (SD): 68.7 (9.7);<br>Participation Rate: 57 of 125 participants (46%) female; 113 of 125 participants (90%) White;<br>Country: USA	Design: RCT<br>Theoretical Framework: Social cognitive, self-management, and pathophysiological theories<br>Major Findings: There were no differences in dyspnea with activities of daily living across groups over 12 months (P= 0.48). Participants in both the Internet and face-to-face program s perceived high levels of support for initiating an exercise program.	Setting: Dyspnea and exercise consultation was conducted in the home of the participants.<br>Staff: Advanced practice nurses conducted the consultations.	Cost: Not addressed.<br>Intervention Adaptation: None identified.<br>Process Fidelity: Not addressed.	Individual: At 12 months, there were no differences in dyspnea during activities of daily living across groups. Exercise behavior, performance, and health-related quality of life did not differ across groups (P > 0.05). Only arm endurance (*P*=0.04) differed across groups. Self-efficacy for managing dyspnea improved for both dyspnea self-management groups when compared with control (*P*=0.06).<br>Setting: Not addressed.
