# Supplementary material for: Web 2.0 Chronic Disease Self-Management for Older Adults: A Systematic Review
Source: J Med Internet Res. 2013 Feb 14;15(2):e35. doi: 10.2196/jmir.2439 (PMC3636299; doi:10.2196/jmir.2439)
Supplement: Supplementary file 4 [file jmir_v15i2e35_app4.pdf]

Multimedia Appendix 4. SQS scores for articles.

	Reach	Efficacy				Adoption		Implementation			Maintenance		SQS	SQS
Author, Year	Representative-ness (0-3)	Design Suitability (1-7)	Data Credibility (0-3)	Evaluation (0-12)	Stats (0-3)	Setting Level (0-4)	Staff Level (0-4)	Web 2.0 Uptake (0-4)	Intervent. Develop (0-3)	Program Integration (0-8)	Setting Level (0-7)	Ind Level (0-3)	Total (1-61)	Percent Score (%)
Nguyen et al, 2008 [47]	3	6	2	10	2	0	4	3	2	5	4	1	42	69%
Soloman, Wagner, & Goes, 2012 [48]	3	6	2	5	3	2	0	1	1	3	2	0	28	46%
Glasgow et al, 2011 [49]	2	6	2	10	2	4	2	4	3	6	2	0	43	70%
Richardson et al, 2010 [50]	3	7	2	10	3	4	4	4	3	6	6	0	52	85%
Nijland et al, 2011 [51]	2	4	1	8	2	0	2	2	3	5	3	3	35	57%
Cudney & Weinert, 2012 [52]	2	5	1	0	1	1	1	3	1	1	0	0	16	26%
Marziali, 2009 [53]	2	2	1	3	0	2	2	2	2	5	3	2	26	43%
Smarr, Shigaki, Donovan, & Hansen, 2011 [54]	1	2	1	5	0	2	2	2	2	7	2	3	29	48%
McMahon et al, 2005 [55]	2	6	2	9	2	0	4	4	0	5	6	3	43	70%
Lorig et al, 2010 [56]	3	6	3	9	3	3	2	4	3	7	6	2	51	84%
Bond et al, 2010 [57]	2	7	2	6	3	4	0	1	1	2	0	2	30	49%
Glasgow et al, 2012 [58]	2	7	3	10	3	4	2	4	2	8	2	2	49	80%
Lorig et al, 2008 [59]	3	6	3	9	3	4	3	4	3	6	6	2	52	85%
Lorig et al, 2012 [60]	2	5	3	6	3	3	2	1	2	5	5	3	40	66%
Nguyen et al, 2012 [61]	3	7	2	8	2	1	3	4	2	7	0	0	39	64%

Mean<br>(SD)	2.33<br>(0.62)	5.47<br>(1.64)	2<br>(0.76)	7.2<br>(2.98)	2.13<br>(1.06)	2.27<br>(1.58)	2.2<br>(1.26)	2.87<br>(1.25)	2<br>(0.93)	5.2<br>(1.93)	3.13<br>(2.26)	1.53<br>(1.25)	38.33<br>(10.43)	63%<br>(18%)
