# Supplementary material for: Web 2.0 Chronic Disease Self-Management for Older Adults: A Systematic Review
Source: J Med Internet Res. 2013 Feb 14;15(2):e35. doi: 10.2196/jmir.2439 (PMC3636299; doi:10.2196/jmir.2439)
Supplement: Supplementary file 5 [file jmir_v15i2e35_app5.pdf]

Author(s), Year	Web Design	Interactivity	Content
Nguyen HQ, Donesky-Cuenco D, Wolpin S, Reinke LF, Benditt JO, Paul SM, Carrieri-Kohlman V; 2008 [47]	The Internet-based (eDSMP) participants submitted real-time information about their symptoms (dyspnea, sputum, sputum purulence, symptoms of a cold, wheezing, and cough) and exercise (mode, duration, and worst dyspnea) via PDA or website. The nurses reviewed uploaded information to provide individualized feedback and reinforcement to participants regarding their use of dyspnea management strategies and exercise progress via email. Automated email alerts were sent to the study nurses based on real-time symptom exacerbation (worsening of symptoms from usual) and exercise decline (not submitting exercise reports for at least 3 consecutive days).	Participants in eDSMP groups communicated with the nurse asynchronously via email.	Web-based education modules written at the 8th grade level or lower using non-digitized audio, pictures and animations. Content reinforced during six weekly chat sessions with participants.
Solomon M, Wagner S, Goes J; 2012 [48]	Personal health portal featuring a suite of interactive health applications. Each week of the study, intervention participants received an email alerting them to the availability of the next health education sessions. Patients review site at their own pace and select the level of complexity of the material.	Asynchronous email communication with their providers using secure email communication function if they require more information.	Progressive series of health education sessions specific to their chronic condition. MyHealth Online users could book doctors' office appointments online, request prescription renewals, and view and pay bills.
Glasgow RE, Christiansen SM, Kurz D, Woolley T, Faber AJ, Estabrooks PA, Strycker L, Toobert D, Dickman J; 2011 [49]	User data entry and management of graphical displays for self-monitoring and laboratory test results. Website included graphical display of clinical measures; moderated discussion forum called "Ask an Expert"; "My Resources" (web links, recipes, handouts); rotating quiz questions; and motivational tips.	Asynchronous communication with provider (IVR computer-based telephone system and e-mail).	Set goals to enhance self-efficacy in 3 areas through website or IVR. Action plans list the specific management goal with individually tailored reasons for working towards the goal, along with barriers and strategies to overcome barriers. Immediate feedback given on success/struggles in meeting goals over 7 day period.
Richardson CR, Buis LR, Janney AW, Goodrich DE, Sen A, Hess ML, Mehari KS, Fortalge LA, Resnick PJ, Zikmund-Fisher BJ, Strecher VJ, Piette JD; 2010 [50]	Website included graphs of participants' walking progress, individually-tailored motivational messages, and weekly calculated goals. The "online community" arm enabled access to read and post messages among online community participants.	Exchanged messages of support and information in an asynchronous online forum (WebCT platform).	The 16 week Internet-mediated walking program included 4 intervention components: (1) uploading pedometers, (2) step-count feedback, (3) individually assigned and gradually incrementing step-count goals, and (4) individually tailored motivational messages. Content in the online community focused on providing social support, encouraging social modeling of successes, and facilitating use of non-community components of the intervention.
Nijland N, van Gemert-Pijnen J, Kelders SM, Brandenburg BJ, Seydel ER; 2011 [51]	Online calendar available to patients for writing down appointments and personal goals. Core features included My Personal Data; Online Monitoring; Email Contact; Online Education; Calendar; and Lifestyle Coach. Patients' self-monitored data (e.g., weight, blood glucose level, blood pressure, and cholesterol) made available to nurses with alerts signaling alarming metabolic values.	Asynchronous email communication between patient and study nurse. Nurses allowed two (n=2) extra consultation sessions per patient to compensate for extra time needed for study participation.	Nurses monitored/set individual goals for patients, added selected lifestyle programs, and highlighted appropriate chapter(s) of e-learning program. Diabetes information and instructions (in accordance with diabetes care standards and protocols in Netherlands), and self-tests to support lifestyle changes.
Cudney S, Weinert C; 2012 [52]	Utilized a format that enhanced health literacy and was visually inviting. Health teaching units were designed using ample white space, sharp contract and large type. Visuals were selected that contributed to the message to be conveyed.	Exchanged messages of support and information in an asynchronous online forum (WebCT platform).	Delivered sequentially over 11 weeks, 9 online health teaching units focused on (1) how to search for and evaluate health information on the Internet and (2) approaches for problem solving and self-managing chronic conditions (mean Gunning Fog Index of health units = 10)
Marziali E; 2009 [53]	Videoconferencing window located in center of screen surrounded by digital photos of participants and facilitators (picture clicked when participants wishes to speak).	Synchronous Internet-based videoconferencing, asynchronous communication included e-mail and threaded discussion forum.	Support group member-facilitator communication for sharing challenges and strategies for managing chronic disease.
Smarr KL, Musser DR, Shigaki CL, Johnson R, Hanson KD, Siva C; 2011 [54]	Simple navigation design of online private and public communities (requiring no more than three mouse clicks to access any activity); high contrast icons on plain background. Site included private component (individualized SM program and leader support) and group component (online "virtual" community).	Synchronous and asynchronous community building features such as Discussion Boards, Email Messaging, Instant Messaging, and News. Members interacted with the leader via online text box augmented by weekly follow-up phone calls to address members' questions, accommodate individual learning styles, and facilitate integration of new concepts with previously learned material. Restricted web support "community" included health professionals (called leaders) as well as participants with RA (called members).	Personalized display of "To Do" lists that guide users through educational modules, weekly homework assignments, and a journal and symptom self-monitoring system. All text written at a 6th grade level.

Author(s), Year	Web Design	Interactivity	Content
McMahon GT, Gomes HE, Hohne SH, Hu TM, Levine BA, Conlin PR; 2005 [55]	Upload of blood pressure and glucose data from monitoring devices to MyCareTeam website.	An internal asynchronous messaging system on website allowed participants to send and receive secure messages to and from the care manager.	Web enabled diabetes education modules with links to other diabetes resources.
Lorig K, Ritter PL, Laurent DD, Plant K, Green M, Blue Bird Jernigan V, Case Siobhan; 2010 [56]	Interactive web based instruction, exercise logs, and medication diaries available through website. Web-based weekly self-management instruction (The Learning Center); web-based bulletin board discussion (Discussion Center); exercise logs, medication diaries and audio relaxation exercises (My Tools); internal messaging center (Post Office); and program tutorial (Help).	Asynchronous communication (e-mail and Discussion Center).	Topics included: overview of self-management and diabetes, introduction to diabetes, understanding glucose, blood pressure, and body mass index, making an action plan, feedback/problem-solving, exercise, difficult emotions, managing stress, weight control, hypoglycemia, depression, relationships with family, working with the health care system, medications, sick days, and future plans.
Bond GE, Burr RL, Wolf FM, Feldt K; 2010 [57]	Website included ability to submit personal log of diabetes SM activities, tailored SM instruction, bulletin board, question and answer forum. User uploaded blood glucose readings, weight changes, blood pressure, and medication data.	Synchronous communication with medical personnel (instant messaging and chat); Asynchronous communication with medical personnel (e-mail with study nurse and bulletin board) and online patient forum (ShareCircle). Weekly online discussion group via instant messaging (MSN Messenger).	Access to a library of articles and sites on diabetes and online advice/counseling from a nurse via email. Weekly educational discussion sessions stemmed from resources published by national diabetes organization.
Glasgow RE, Kurz D, King D, Dickman JM, Faber AJ, Halterman E, Woolley T, Toobert DJ, Strycker LA, Estabrooks PA, Osuna D, Ritzwoller D; 2012 [58]	See Glasgow et al., 2011 [49]	See Glasgow et al., 2011 [49]	See Glasgow et al., 2011 [49]
Lorig KR, Ritter PL, Laurent DD, Plant K; 2008 [59]	Availability of exercise logs and medication diaries through website. Interactive web based instruction also available on website. Web-based instruction (The Learning Center); web-based bulletin board discussion (The Discussion Center); exercise logs, medication diaries and tailored exercise programs (My Tools).	Asynchronous communication (e-mail and Discussion Center).	Learning Center content included design of individual exercise programs; use of cognitive symptom management such as relaxation; visualization; distraction and self-talk; methods for managing negative emotions; aspects of patient-provider communication; healthy eating fatigue management; action planning; feedback; and methods for problem solving.
Lorig K, Ritter PL, Plant K, Laurent DD, Kelly P, Rowe S; 2012 [60]	Availability of exercise logs, and medication diaries through website. Interactive web based instruction also available through website. Web-based weekly self-management instruction (The Learning Center); web-based bulletin board discussion (Discussion Center); exercise logs, medication diaries and audio relaxation exercises (My Tools); internal messaging center (Post Office); and program tutorial (Help).	Asynchronous communication (e-mail, and the Discussion Center).	Information about how to deal with various aspects of chronic disease that is self-tailored to each participant.
Nguyen HQ, Donesky D, Reinke LF, Wolpin S, Chyall L, Benditt JO, Paul SM, Carrieri-Kohlman V; 2012 [61]	eDSMP participants submitted real-time information about their symptoms (dyspnea, sputum, sputum purulence, symptoms of a cold, wheezing, and cough) and exercise (mode, duration, and worst dyspnea) via PDA or website. Nurses reviewed uploaded information to provide individualized feedback	Participants in eDSMP groups communicated with nurse asynchronously via email and had access to six 1-hour live group chat sessions.	Web-based education modules written at the 8th grade level or lower using non-digitized audio, pictures and animations. Content reinforced during six weekly chat sessions with participants. Participants received education on shortness of breath (SOB), breathing strategies to reduce SOB, exercise and SOB, modifying activities to reduce SOB, coping with SOB and stress, and medications to manage SOB and COPD flare-ups.
